# Altered functional association and couplings: Effective diagnostic neuromarkers for Alzheimer’s disease

**DOI:** 10.3389/fnagi.2022.1009632

**Published:** 2022-10-13

**Authors:** Chongyi Zhao, Meiling Chen, Zhiyong Ding, Chunyan Liu, Xiaomei Wu

**Affiliations:** ^1^Faculty of Life Science and Technology, Kunming University of Science and Technology, Kunming, China; ^2^Department of Gynecology, The First People’s Hospital of Yunnan Province, Kunming University of Science and Technology, Kunming, China; ^3^Department of Clinical Psychology, The First People’s Hospital of Yunnan Province, Kunming University of Science and Technology, Kunming, China; ^4^Department of Medical Imaging, Qujing Maternal and Child Health Care Hospital, Kunming University of Science and Technology, Qujing, China; ^5^Department of Neurology, Xuanwu Hospital, Capital Medical University, Beijing, China; ^6^Beijing Key Laboratory of Neuromodulation, Beijing, China

**Keywords:** Alzheimer’s disease, association mapping, functional connectivity, disconnection, biomarker

## Abstract

Alzheimer’s disease (AD) is a common neurodegenerative disorder causing dementia in the elderly population. Functional disconnection of brain is considered to be the main cause of AD. In this study, we applied a newly developed association (Asso) mapping approach to directly quantify the functional disconnections and to explore the diagnostic effects for AD with resting-state functional magnetic resonance imaging data from 36 AD patients and 42 age-, gender-, and education-matched healthy controls (HC). We found that AD patients showed decreased Asso in left dorsoanterior insula (INS) while increased functional connections of INS with right medial prefrontal cortex (MPFC) and left posterior cingulate cortex (PCC). The changed Asso and functional connections were closely associated with cognitive performances. In addition, the reduced Asso and increased functional connections could serve as effective neuromarkers to distinguish AD patients from HC. Our research provided new evidence for functional disconnections in AD and demonstrated that functional disconnections between cognition-memory networks may be potential early biomarkers for AD.

## Introduction

Alzheimer’s disease (AD) is a typical neurodegenerative disorder and the most common cause of dementia with a core symptom of progressive cognitive declines (Ballard et al., 2011). Almost 15% of old adults over 65 years old have mild cognitive impairment and more than half of these people become to AD within 5 years (Farlow, 2009). AD is mainly manifested with loss of memory, language, attention, executive, and perceptive functions (Petersen et al., 1999; Dubois et al., 2007; Petersen, 2009; Dubois et al., 2010; Sheline and Raichle, 2013). With the disease progresses, AD patients gradually lose their ability to take care of themselves and can only rely on caregivers (Dubois et al., 2010). AD has become one of the most serious social and economic burdens on patients and their families. Thus, identification of early detection and diagnosis biomarkers for AD provides an important buffer for developing effective treatments.

The cortical atrophy, especially the hippocampal atrophy is a hallmark of AD (Jack et al., 2000; Fox and Schott, 2004; Apostolova et al., 2012). However, emerging evidence has demonstrated that AD is not only a local lesion but a brain disorder with functional disconnection (de Vos et al., 2018; Hojjati et al., 2019). The disconnections within or between brain functional networks are primary characteristics of AD in spite of a few increased connections reported (Greicius et al., 2004; Wang et al., 2006; Agosta et al., 2012; Wu H. et al., 2016; Zhan et al., 2016; Yu et al., 2021; Liu et al., 2022). A recent study found that the large-scale decoupling of fronto-temporal networks associated with cognitive decline precedes structural deficit in individuals with mild cognitive impairment and may represent a potential biomarker for disease progression (Broadhouse et al., 2021). Thus, to reveal the functional dissociations of brain in AD patients may facilitate establishing an early diagnosis and prevention target. The previous studies mainly used functional connectivity strength (FCS), i.e., degree centrality method to measures functional associations (Wu H. et al., 2016; Liu C. et al., 2018; Liu et al., 2022; Cheng et al., 2022a), but FCS only counts the connected edges of the seed voxel with other voxels but neglect whether or not its connected neighborhoods are also directly interconnected to each other since the interconnective state of neighboring voxels will degrade the importance of the seed voxel in information transition. To overcome the limitation of FCS, a threshold-free method of association mapping (Asso) was recently developed to quantitatively characterize the brain association ability at the voxel level (Chen et al., 2021). Asso mapping approach identified a significant gradient distribution with high Asso values in association cortical networks while low Asso values in visual and limbic networks. In addition, Asso mapping can better reveal the aging effects than FCS. Therefore, Asso mapping is particularly useful to reveal the early functional dissociations in AD and open a new avenue to reveal early biomarker for onset of AD.

Using the newly developed Asso approach, the present study first revealed the functional dissociations in AD. Then, the functional connectivity (FC) analysis was used to identify disrupted functional couplings with other brain areas. Next, support vector machine (SVM) was applied to test whether the changed Asso and FC could serve as biomarkers to distinguish AD from healthy controls (HC). Finally, correlation analyses were performed to establish the relationships between changed Asso or FC and cognitive performances.

## Materials and methods

### Subjects

Thirty-six drug-free patients with AD and 42 age-, gender-, and education-matched HC were recruited from Xuanwu Hospital, Capital Medical University, China. AD was diagnosed by 2 trained senior neurologists with a structured clinical interview. The inclusion criteria were as follows: (1) right-handed with age from 50 to 85 years, (2) meeting the diagnosis criteria of 2011 National Institute on Aging AD (NIA-AA) guidelines, (3) clinical dementia rating (CDR) (score of 0.5 or 1.0, 4) geriatric depression scale (GDS) ≤ 8 and hachinski ischemic scale (HIS) < 4. The exclusion criteria include (1) complications with other severe heart, liver, lung, kidney, or neurological diseases; (2) currently taking benzodiazepines or having a history of drug abuse; (3) comorbid with other mental disorders. The written informed consents were provided and obtained by all participants or their families. The clinical symptom severity for AD patients was assessed using the Alzheimer’s disease assessment scale-cognitive subscale (ADAS-Cog) and CDR scale. In addition, the Mini-Mental State Examination (MMSE) and Montreal Cognitive Assessment (MoCA) were also employed to evaluate the cognitive performances for all the participants. This study was approved by the local medical research ethics committee at Xuanwu hospital, Capital Medical University, China. The detailed information for all the participants can be found in our previous study (Liu et al., 2022).

### Resting-state fMRI data acquisition

The functional MR images were acquired on a clinical Siemens 3.0 T MRI scanner (Siemens, Erlangen, Germany) with an echo planar imaging sequence. Subjects were instructed to keep their eyes closed and to relax during scanning. The foam padding was used to reduce head motion. The acquisition parameters were: repetition time (TR) = 2,000 ms, echo time (TE) = 40 ms, flip angle (FA) = 90°, matrix size = 64 × 64, slices = 28, thickness/gap = 4.0/1.0 mm, voxel size = 3.75 × 3.75 × 4 mm^3^, 240 volumes.

### Resting-state fMRI data preprocessing

The resting-state fMRI data was preprocessed with the following steps: (1) discard the first 10 volumes to allow for magnetization equilibrium; (2) head motion correction by realigning all the volumes to the first volume. The participants with excessive head movements above one voxel were excluded; (3) normalize the fMRI images to the Montreal Neurological Institute (MNI) template and re-sampled to 3-mm isotropic voxels; (4) all the images were detrended and filtered with temporal band-path (0.01–0.1 Hz); (5), the Friston-24 head motion parameters, white matter, cerebrospinal fluid, and global mean signals regressed out. To further eliminate head motion effects, the bad images (before 2 time points and after 1 time points) exceeding the pre-set criterion (frame displacement < 0.5) for excessive motion were scrubbed using linear interpolation (Cheng et al., 2021, 2022b; Pang et al., 2022a,b).

### Association index calculation

The Asoo for a specific voxel was calculated as the total number of functional connections of this voxel to all other voxels minus that of interconnected functional connections of all the other voxels. The functional connections were considered only the connectivity strength above 0.25. The details for Asso calculation are the following. First, the whole-brain functional connectivities using Pearson’s correlation coefficients were calculated for a specific voxel. Then, a predefined threshold of 0.25 was used to identify the number (*N*) of functional connectivities with higher coefficients than this threshold. Next, the number (*K*) of functional connectivities higher than 0.25 among the above identified voxels was further calculated. Finally, the Asoo for this specific voxel was computed as the following formula. Finally, a whole-brain Asoo map for each subject was obtained using the same procedures for the voxels of the whole brain. The z-transformation was applied to whole brain Asso map to improve normality and smoothed with 6 mm full-width-at-half-maximum (FWHM) Gaussian kernel for statistical analysis. A two-sample *t*-test with age, gender, and education as covariates was used to identify differences between AD and HC. The significance was determined with a cluster-level corrected threshold of *p* < 0.05 (cluster-forming threshold at voxel-level *p* < 0.001).


(1) $$A\,s\,s\,o=\frac{N\times \left(N-1\right)-2\times K}{2}$$

### Functional connectivity analysis

To calculate FC, the normalized functional images were smoothed using a 6 mm FWHM Gaussian kernel, and then, detrending, filtering, and regression were performed to finish the fMRI data preprocessing. For FC analysis, the seed region was defined as the brain areas showing difference in Asso index. The mean time series was then extracted for the seed region, and the FC was measured using Pearson’s correlations between the averaged time series of the seed region and voxels in the rest of the brain. Finally, the Fisher’s z transform was applied to normalize the FC maps. To identify FC difference, a two sample *t*-test with age, gender, and educational as covariates was performed between AD and HC. The significance was determined with a cluster-level corrected threshold of *p* < 0.05 (cluster-forming threshold at voxel-level *p* < 0.001).

### Functional characterization with BrainMap database

To characterize the behavioral associations of the brain areas showing differences in Asso and FC, functional characterization of the brain areas were performed using the behavioral domain analysis on the BrainMap database.^1^ The behavioral domain analysis including 5 behavioral domains and 51 behavioral sub-domains was determined using forward inferences. The significance level was established using a binomial test [*p* < 0.05 corrected for multiple comparisons using false discovery rate (FDR)] (Bzdok et al., 2013). The detailed procedures for functional characterization have been described in our previous studies (Eickhoff et al., 2009; Clos et al., 2013; Wang et al., 2015, 2017a,^2019^; Wang J. et al., 2020).

### Multivariate pattern classification using support vector machine

To explore whether changed Asso and FC could effectively distinguish AD from HC, a linear SVM and a leave-one-out cross-validation were used to estimate the generalization. The classification results were evaluated using index of accuracy, sensitivity, specificity, and the area under the curve (AUC) value.

### Correlation analysis

To explore whether the changed Asso or FC was associated with the clinical or cognitive performances, the correlation analyses were performed and the significant level was set at *p* < 0.05 corrected with FDR-BH method.

## Results

### Demographic and behavioral information

A chi-square was used to determine gender difference and two-sample *t*-tests were applied to determine the differences in age, education, MMSE, and MoCA. There are no significant differences in gender (*p* = 0.097), age (*p* = 0.097), and education (*p* = 0.29). Compared to HC, AD patients showed significantly lower MMSE (*p* = 1.08 × 10^–19^) and MoCA (*p* = 4.02 × 10^–27^) (Table 1).

**TABLE 1:** Demographics and clinical information.

Subjects	AD	HC	*p-*value
Number of subjects	36	42	
Gender (male: female)	21/15	16/26	0.097
Age (mean ± SD)	68.84 ± 7.84	66.07 ± 6.79	0.097
Years of education (mean ± SD)	10.84 ± 3.11	11.62 ± 3.32	0.29
MMSE (mean ± SD)	21.3 ± 3.36	28.24 ± 1.43	1.08 × 10^–19^
MoCA (mean ± SD)	16.92 ± 3.08	26.95 ± 2.01	4.02 × 10^–27^
Duration of illness (years)	3.08 ± 1.92		
ADAS-Cog	18.81 ± 7.49		

A Pearson chi-squared test was used for gender comparison. Two-sample *t*-tests were used for age, years of education, MMSE, and MoCA comparisons. MMSE, Mini-mental State Examination; MoCA, Montreal Cognitive Assessment; ADAS-Cog, Alzheimer’s Disease Assessment Scale-Cognitive Subscale test; AD, Alzheimer’s Disease; HC, healthy control.

### Changed association ability

**FIGURE 1:**
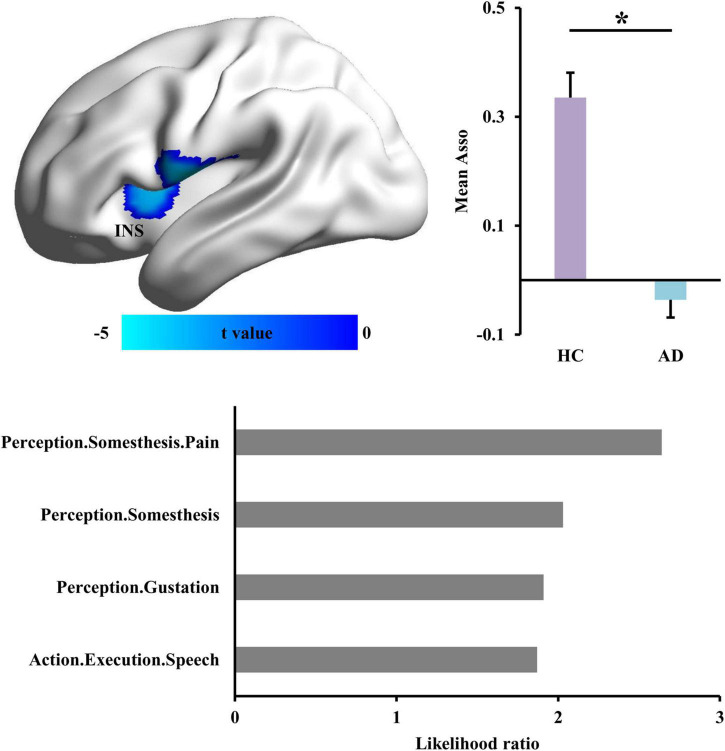
Changed functional associations in Alzheimer’s disease (AD) patients. The whole brain voxel-wise association ability (Asso) mapping was applied to identify the difference in AD patients compared with healthy controls. The reduced Asso in left dorsal anterior insula (INS) was found in AD. The functional characterization of INS using BrainMap database found that this area is mainly involved in somesthesis perception and speech. *Represents a significant difference.

To identify the disrupted association ability in AD patients, Asso analysis identified significantly decreased Asso values in left insula (INS) in AD patients (Figure 1 and Table 2). Functional characterization of left INS revealed that this area is mainly involved in pain and speech processing (Figure 1).

**TABLE 2:** Regions with changed association index (Asso) and functional connectivities (FC) in patients with Alzheimer’s disease compared to healthy controls.

Brain regions	L/R	Peak MNI coordinates			*t*-values
		X	Y	Z	
Insula	L	−51	−9	12	−4.81
Medial prefrontal cortex	R	12	57	9	4.79
Posterior cingulate cortex	L	−6	−48	18	4.62

Two-sample *t*-tests were used to identify changed functional association (Asso) and resting-state functional connectivity (FC) between Alzheimer’s disease and healthy controls. MNI, Montreal Neurological Institute; L, left hemisphere; R, right hemisphere.

### Functional connectivity analysis

**FIGURE 2:**
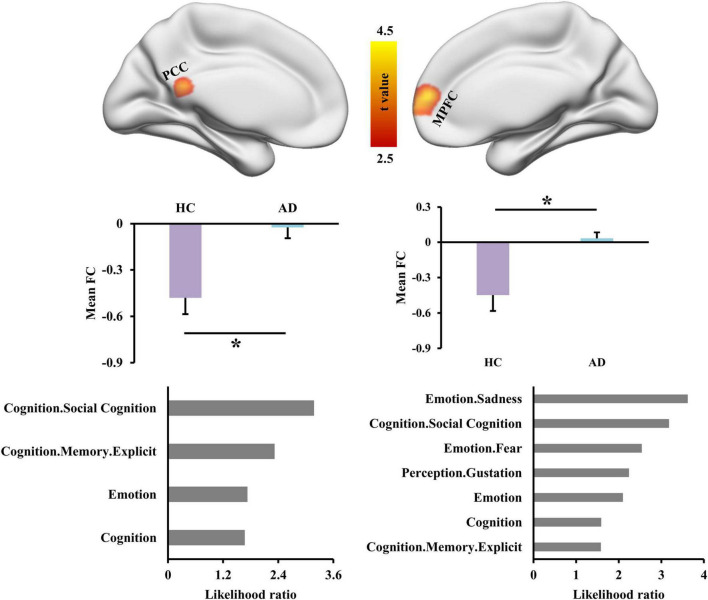
Altered functional connectivities of left dorsal anterior insula (INS) in Alzheimer’s disease (AD) patients. Whole brain voxel-wise functional connectivity analysis identified significantly increased functional couplings between INS and posterior cingulate cortex (PCC) and medial prefrontal cortex (MPFC) in AD patients compared to healthy controls. The functional characterization of PCC found that this area is mainly involved in social cognition, memory, emotion, and cognition. The MPFC was also found to mainly participate in sadness, social cognition, fear, gustation, cognition, and memory. *Represents a significant difference.

With left INS as seed region, whole brain FC analysis identified increased FCS in left posterior cingulate cortex (PCC) and right medial prefrontal cortex (MPFC) in AD patients compared to HC (Figure 2 and Table 2). Functional characterization of PCC revealed that this area is mainly involved in social cognition, explicit memory, emotion, and cognition. Functional characterization of MPFC found that this area is mainly involved in sadness, social cognition, fear, cognition, and explicit memory (Figure 2).

### Classification results

**FIGURE 3:**
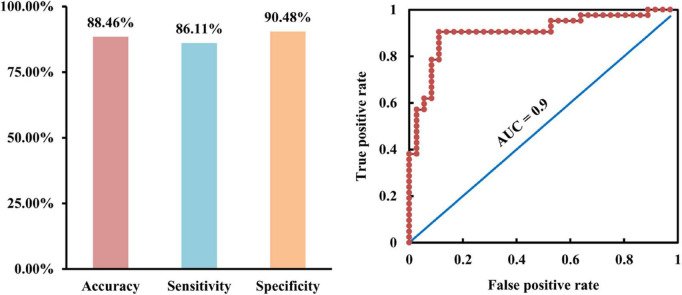
Classification of Alzheimer’s disease (AD) patients from healthy controls using support vector machine (SVM). With the changed association ability and functional connectivities as features, SVM was used and achieved accuracy of 88.46%, sensitivity of 86.11%, specificity of 90.48%, and area under curve (AUC) of 0.9 for distinguishing AD from healthy controls.

To test whether the changed Asso and FC could serve as neuromarkers to distinguish AD patients from HC, SVM was used and achieved an accuracy of 88.46%, sensitivity of 86.11%, specificity of 90.48%, and AUC = 0.9 for AD classification (Figure 3).

### Correlation results

**FIGURE 4:**
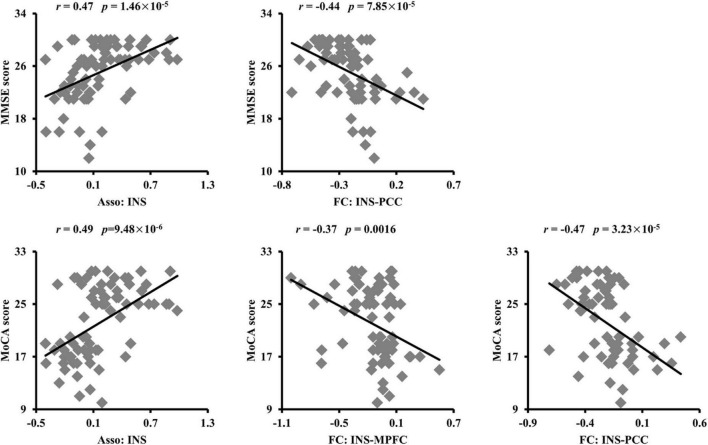
Significant correlations between the association ability, functional connectivities, and cognitive performances. The association ability (Asso) of left dorsal anterior insula (INS) was positively correlated with Mini-Mental State Examination (MMSE) and Montreal Cognitive Assessment (MoCA) scores. The functional connectivity between INS and posterior cingulate cortex (PCC) was negatively correlated with MMSE. The functional connectivities between INS and PCC, medial prefrontal cortex (MPFC) were also negatively correlated with MoCA scores.

To establish the relationship between neural measurements and behavioral performances, Pearson’s correlation analyses were performed. The significantly positive correlation and negative correlation between Asso values in INS, FC of INS with PCC and MMSE scores were found, respectively (Figure 4). In addition, positive correlation between Asso values of INS and MoCA, negative correlations between FCs of INS with PCC, MPFC, and MoCA were also found (Figure 4).

## Discussion

In the present study, we employed a newly proposed method to directly investigate the functional dissociations in patients with AD. The decreased Asso ability in insula was found suggesting the disrupted functional integration of this area in AD patients. The FC analyses revealed increased connectivity of INS with PCC and MPFC in AD patients. Moreover, the changed Asso and FC were closely associated with cognitive performances and could serve as effective biomarkers to distinguish AD from HC. Our findings provide new evidence for disconnection in AD and highlight the important role of insula in neuropathology of AD.

The insular cortex is a hub in the brain for integrating cognition, memory, attention, emotion, and sensory (Craig, 2011; Simmons et al., 2013), and functional impairments have been widely reported in brain disorders as well as in AD patients (Blanc et al., 2014; Lin et al., 2017; Sun et al., 2018; Wang C. et al., 2018; Wang Y. et al., 2018; Wang L. et al., 2020; Fathy et al., 2020; Cheng et al., 2022b). A recent meta-analysis demonstrated that the insula is a common neurobiological substrate for mental illness (Goodkind et al., 2015). All these studies indicated that the important role of insula in the neuropathology of brain diseases. Insula is also a structurally and functionally heterogeneous area. The dorsal anterior insula is mainly involved in cognitive control. The ventral anterior insula primarily participates in emotion processing, and the posterior insula is related to perception of internal states (Deen et al., 2011). In our study, we found that AD patients showed decreased functional associations in dorsal anterior insula suggesting that functional dissociations in cognition related subregion of insula may be an early neuromarkers for cognitive deficits in AD. The conclusion is supported by the correlation analyses which found significant associations of AI values in dorsal anterior insula with cognitive performances in our study. In a word, these results demonstrated that Asso index is a useful measure for early characterization of the onset of AD and the dorsal anterior insula is a key target for early prevention of AD.

The dorsal anterior insula, a part of salience network, acts as an interface between internal and external stimuli and plays an important role in flexibly and dynamically switching between task positive network, i.e., executive control network and task negative network, default mode network (DMN) (Menon and Uddin, 2010; Menon, 2011; Liang et al., 2016; Wang J. et al., 2018). Thus, dorsal anterior insula is important to maintain the dynamic balance of brain. DMN mainly includes PCC, MPFC, angular gyrus, dorsolateral prefrontal cortex, and medial temporal lobule and thus is closely related to social cognition, memory, and emotion processing (Buckner et al., 2008; Raichle, 2015; Wang et al., 2017b,^2019^). In our study, we found AD patients showed increased functional connections with PCC and MPFC which are two core areas in DMN (Andrews-Hanna et al., 2010). Our finding is supported by a previous study which used seed-based FC analysis of insular subregions and also identified increased functional connections of dorsal anterior insula with DMN related brain regions in AD patients compared to HC (Liu X. et al., 2018). The increased FC between dorsal anterior insula and DMN indicated disrupted dynamic balance between internal and external states of brain and may reflect compensation for cognitive declines in AD patients. This is line with the correlation analyses results that functional connectivities between dorsal anterior insula and PCC, MPFC were negatively correlated with MMSE and MoCA scores in this study. Together with the classification results, our findings of decreased functional association ability of dorsal anterior insula and functional connectivities of dorsal anterior insula with PCC and MPFC provide new biomarkers for AD.

## Conclusion

In conclusion, the present study assessed the alteration of functional dissociation and couplings in patients with AD. The decreased functional associations of dorsal anterior insula and functional couplings with DMN were found in AD patients. The abnormal functional association and connectivities of insula were significantly associated with cognitive performances. The identified insula-DMN circuits may be new biomarkers for early diagnosis and prevention targets for AD.

## Data availability statement

The raw data supporting the conclusions of this article will be made available by the authors, without undue reservation.

## Ethics statement

The studies involving human participants were reviewed and approved by the Xuanwu Hospital, Capital Medical University. The patients/participants provided their written informed consent to participate in this study.

## Author contributions

XW and CL designed and supervised the study and edited the manuscript. CL collected the data. CZ and ZD analyzed the data. CZ, ZD, and MC wrote the manuscript. All authors discussed the results and commented on the manuscript.

## Footnotes

1
www.brainmap.org

## References

[B1] Agosta F. Pievani M. Geroldi C. Copetti M. Frisoni G. B. Filippi M. (2012). Resting state fMRI in Alzheimer’s disease: beyond the default mode network. *Neurobiol. Aging* 33 1564–1578. 10.1016/j.neurobiolaging.2011.06.007 21813210

[B2] Andrews-Hanna J. R. Reidler J. S. Sepulcre J. Poulin R. Buckner R. L. (2010). Functional-anatomic fractionation of the brain’s default network. *Neuron* 65 550–562. 10.1016/j.neuron.2010.02.005 20188659 PMC2848443

[B3] Apostolova L. G. Green A. E. Babakchanian S. Hwang K. S. Chou Y. Y. Toga A. W. (2012). Hippocampal atrophy and ventricular enlargement in normal aging, mild cognitive impairment (MCI), and Alzheimer disease. *Alzheimer Dis. Assoc. Disord.* 26 17–27. 10.1097/WAD.0b013e3182163b62 22343374 PMC3286134

[B4] Ballard C. Gauthier S. Corbett A. Brayne C. Aarsland D. Jones E. (2011). Alzheimer’s disease. *Lancet* 377 1019–1031. 10.1016/S0140-6736(10)61349-9 21371747

[B5] Blanc F. Noblet V. Philippi N. Cretin B. Foucher J. Armspach J.-P. (2014). Right anterior insula: core region of hallucinations in cognitive neurodegenerative diseases. *PLoS One* 9:e114774. 10.1371/journal.pone.0114774 25479196 PMC4257732

[B6] Broadhouse K. M. Winks N. J. Summers M. J. (2021). Fronto-temporal functional disconnection precedes hippocampal atrophy in clinically confirmed multi-domain amnestic mild cognitive impairment. *EXCLI J.* 20 1458–1473. 10.17179/excli2021-4191 34737688 PMC8564906

[B7] Buckner R. L. Andrews-Hanna J. R. Schacter D. L. (2008). The brain’s default network: anatomy, function, and relevance to disease. *Ann. N.Y. Acad. Sci.* 1124 1–38. 10.1196/annals.1440.011 18400922

[B8] Bzdok D. Laird A. R. Zilles K. Fox P. T. Eickhoff S. B. (2013). An investigation of the structural, connectional, and functional subspecialization in the human amygdala. *Hum. Brain Mapp.* 34 3247–3266. 10.1002/hbm.22138 22806915 PMC4801486

[B9] Chen K. Wang L. Zeng J. Chen A. Gao Z. Wang J. (2021). Voxel-wise quantitative mapping of the brain association ability. *Front. Neurosci.* 15:746894. 10.3389/fnins.2021.746894 34720865 PMC8555663

[B10] Cheng B. Roberts N. Zhou Y. Wang X. Li Y. Chen Y. (2022a). Social support mediates the influence of cerebellum functional connectivity strength on postpartum depression and postpartum depression with anxiety. *Transl. Psychiatry* 12:54. 10.1038/s41398-022-01781-9 35136017 PMC8826948

[B11] Cheng B. Wang X. Roberts N. Zhou Y. Wang S. Deng P. (2022b). Abnormal dynamics of resting-state functional activity and couplings in postpartum depression with and without anxiety. *Cerebr. Cortex* 2022:bhac038. 10.1093/cercor/bhac038 35174863

[B12] Cheng B. Zhou Y. Kwok V. P. Y. Li Y. Wang S. Zhao Y. (2021). Altered functional connectivity density and couplings in postpartum depression with and without anxiety. *Soc. Cogn. Affect. Neurosci.* 17 756–766. 10.1093/scan/nsab127 34904174 PMC9340108

[B13] Clos M. Amunts K. Laird A. R. Fox P. T. Eickhoff S. B. (2013). Tackling the multifunctional nature of Broca’s region meta-analytically: co-activation-based parcellation of area 44. *NeuroImage* 83 174–188. 10.1016/j.neuroimage.2013.06.041 23791915 PMC4791055

[B14] Craig A. D. (2011). Significance of the insula for the evolution of human awareness of feelings from the body. *Ann. N.Y. Acad. Sci.* 1225 72–82. 10.1111/j.1749-6632.2011.05990.x 21534994

[B15] de Vos F. Koini M. Schouten T. M. Seiler S. van der Grond J. Lechner A. (2018). A comprehensive analysis of resting state fMRI measures to classify individual patients with Alzheimer’s disease. *NeuroImage* 167 62–72. 10.1016/j.neuroimage.2017.11.025 29155080

[B16] Deen B. Pitskel N. B. Pelphrey K. A. (2011). Three systems of insular functional connectivity identified with cluster analysis. *Cerebr. Cortex* 21 1498–1506. 10.1093/cercor/bhq186 PMC3116731 21097516

[B17] Dubois B. Feldman H. H. Jacova C. Cummings J. L. Dekosky S. T. Barberger-Gateau P. (2010). Revising the definition of Alzheimer’s disease: a new lexicon. *Lancet Neurol.* 9 1118–1127. 10.1016/S1474-4422(10)70223-4 20934914

[B18] Dubois B. Feldman H. H. Jacova C. Dekosky S. T. Barberger-Gateau P. Cummings J. (2007). Research criteria for the diagnosis of Alzheimer’s disease: revising the NINCDS-ADRDA criteria. *Lancet Neurol.* 6 734–746. 10.1016/S1474-4422(07)70178-3 17616482

[B19] Eickhoff S. B. Laird A. R. Grefkes C. Wang L. E. Zilles K. Fox P. T. (2009). Coordinate-based activation likelihood estimation meta-analysis of neuroimaging data: a random-effects approach based on empirical estimates of spatial uncertainty. *Hum. Brain Mapp.* 30 2907–2926. 10.1002/hbm.20718 19172646 PMC2872071

[B20] Farlow M. (2009). Treatment of mild cognitive impairment (MCI). *Curr. Alzheimer Res.* 6 362–367. 10.2174/156720509788929282 19689235

[B21] Fathy Y. Y. Hepp D. H. de Jong F. J. Geurts J. J. G. Foncke E. M. J. Berendse H. W. (2020). Anterior insular network disconnection and cognitive impairment in Parkinson’s disease. *NeuroImage Clin.* 28:102364. 10.1016/j.nicl.2020.102364 32781423 PMC7417948

[B22] Fox N. C. Schott J. M. (2004). Imaging cerebral atrophy: normal ageing to Alzheimer’s disease. *Lancet* 363 392–394. 10.1016/S0140-6736(04)15441-X 15074306

[B23] Goodkind M. Eickhoff S. B. Oathes D. J. Jiang Y. Chang A. Jones-Hagata L. B. (2015). Identification of a common neurobiological substrate for mental illness. *JAMA Psychiatry* 72 305–315. 10.1001/jamapsychiatry.2014.2206 25651064 PMC4791058

[B24] Greicius M. D. Srivastava G. Reiss A. L. Menon V. (2004). Default-mode network activity distinguishes Alzheimer’s disease from healthy aging: evidence from functional MRI. *Proc. Natl. Acad. Sci. U.S.A.* 101 4637–4642. 10.1073/pnas.0308627101 15070770 PMC384799

[B25] Hojjati S. H. Ebrahimzadeh A. Babajani-Feremi A. (2019). Identification of the early stage of Alzheimer’s disease using structural MRI and resting-state fMRI. *Front. Neurol.* 10:904. 10.3389/fneur.2019.00904 31543860 PMC6730495

[B26] Jack C. R. Petersen R. C. Xu Y. O’brien P. Smith G. E. Ivnik R. J. (2000). Rates of hippocampal atrophy correlate with change in clinical status in aging and AD. *Neurology* 55 484–490. 10.1212/wnl.55.4.484 10953178 PMC2724764

[B27] Liang X. Zou Q. He Y. Yang Y. (2016). Topologically reorganized connectivity architecture of default-mode, executive-control, and salience networks across working memory task loads. *Cerebr. Cortex* 26 1501–1511. 10.1093/cercor/bhu316 25596593 PMC4785946

[B28] Lin F. Ren P. Lo R. Y. Chapman B. P. Jacobs A. Baran T. M. (2017). Insula and inferior frontal gyrus’ activities protect memory performance against Alzheimer’s disease pathology in old age. *J. Alzheimers Dis.* 55 669–678. 10.3233/JAD-160715 27716674 PMC5531269

[B29] Liu C. Han T. Xu Z. Liu J. Zhang M. Du J. (2022). Modulating gamma oscillations promotes brain connectivity to improve cognitive impairment. *Cerebr. Cortex* 32 2644–2656. 10.1093/cercor/bhab371 34751749

[B30] Liu C. Wang J. Hou Y. Qi Z. Wang L. Zhan S. (2018). Mapping the changed hubs and corresponding functional connectivity in idiopathic restless legs syndrome. *Sleep Med.* 45 132–139. 10.1016/j.sleep.2017.12.016 29680421

[B31] Liu X. Chen X. Zheng W. Xia M. Han Y. Song H. (2018). Altered functional connectivity of insular subregions in Alzheimer’s disease. *Front. Aging Neurosci.* 10:676624. 10.3389/fnins.2021.676624 PMC5905235 29695961

[B32] Menon V. (2011). Large-scale brain networks and psychopathology: a unifying triple network model. *Trends Cogn. Sci.* 15 483–506. 10.1016/j.tics.2011.08.003 21908230

[B33] Menon V. Uddin L. Q. (2010). Saliency, switching, attention and control: a network model of insula function. *Brain Struct. Funct.* 214 655–667. 10.1007/s00429-010-0262-0 20512370 PMC2899886

[B34] Pang Y. Wei Q. Zhao S. Li N. Li Z. Lu F. (2022a). Enhanced default mode network functional connectivity links with electroconvulsive therapy response in major depressive disorder. *J. Affect. Disord.* 306 47–54. 10.1016/j.jad.2022.03.035 35304230

[B35] Pang Y. Zhao S. Li Z. Li N. Yu J. Zhang R. (2022b). Enduring effect of abuse: childhood maltreatment links to altered theory of mind network among adults. *Hum. Brain Mapp.* 43 2276–2288. 10.1002/hbm.25787 35089635 PMC8996351

[B36] Petersen R. C. (2009). Early diagnosis of Alzheimer’s disease: is MCI too late? *Curr. Alzheimer Res.* 6 324–330. 10.2174/156720509788929237 19689230 PMC3098139

[B37] Petersen R. C. Smith G. E. Waring S. C. Ivnik R. J. Tangalos E. G. Kokmen E. (1999). Mild cognitive impairment: clinical characterization and outcome. *Arch. Neurol.* 56 303–308. 10.1001/archneur.56.3.303 10190820

[B38] Raichle M. E. (2015). The brain’s default mode network. *Annu. Rev. Neurosci.* 38 433–447. 10.1146/annurev-neuro-071013-014030 25938726

[B39] Sheline Y. I. Raichle M. E. (2013). Resting state functional connectivity in preclinical Alzheimer’s disease. *Biol. Psychiatry* 74 340–347. 10.1016/j.biopsych.2012.11.028 23290495 PMC3537262

[B40] Simmons W. K. Avery J. A. Barcalow J. C. Bodurka J. Drevets W. C. Bellgowan P. (2013). Keeping the body in mind: insula functional organization and functional connectivity integrate interoceptive, exteroceptive, and emotional awareness. *Hum. Brain Mapp.* 34 2944–2958. 10.1002/hbm.22113 22696421 PMC6870113

[B41] Sun H. Luo L. Yuan X. Zhang L. He Y. Yao S. (2018). Regional homogeneity and functional connectivity patterns in major depressive disorder, cognitive vulnerability to depression and healthy subjects. *J. Affect. Disord.* 235 229–235. 10.1016/j.jad.2018.04.061 29660636

[B42] Wang C. Wu H. Chen F. Xu J. Li H. Li H. (2018). Disrupted functional connectivity patterns of the insula subregions in drug-free major depressive disorder. *J. Affect. Disord.* 234 297–304. 10.1016/j.jad.2017.12.033 29587165

[B43] Wang J. Becker B. Wang L. Li H. Zhao X. Jiang T. (2019). Corresponding anatomical and coactivation architecture of the human precuneus showing similar connectivity patterns with macaques. *NeuroImage* 200 562–574. 10.1016/j.neuroimage.2019.07.001 31276799

[B44] Wang J. Wei Q. Bai T. Zhou X. Sun H. Becker B. (2017a). Electroconvulsive therapy selectively enhanced feedforward connectivity from fusiform face area to amygdala in major depressive disorder. *Soc. Cogn. Affect. Neurosci.* 12 1983–1992. 10.1093/scan/nsx100 28981882 PMC5716231

[B45] Wang J. Wei Q. Wang L. Zhang H. Bai T. Cheng L. (2018). Functional reorganization of intra- and internetwork connectivity in major depressive disorder after electroconvulsive therapy. *Hum. Brain Mapp.* 39 1403–1411. 10.1002/hbm.23928 29266749 PMC6866547

[B46] Wang J. Xie S. Guo X. Becker B. Fox P. T. Eickhoff S. B. (2017b). Correspondent functional topography of the human left inferior parietal lobule at rest and under task revealed using resting-state fMRI and coactivation based parcellation. *Hum. Brain Mapp.* 38 1659–1675. 10.1002/hbm.23488 28045222 PMC6867154

[B47] Wang J. Yang Y. Fan L. Xu J. Li C. Liu Y. (2015). Convergent functional architecture of the superior parietal lobule unraveled with multimodal neuroimaging approaches. *Hum. Brain Mapp.* 36 238–257. 10.1002/hbm.22626 25181023 PMC4268275

[B48] Wang J. Yang Y. Zhao X. Zuo Z. Tan L.-H. (2020). Evolutional and developmental anatomical architecture of the left inferior frontal gyrus. *NeuroImage* 222:117268. 10.1016/j.neuroimage.2020.117268 32818615

[B49] Wang L. Wei Q. Wang C. Xu J. Wang K. Tian Y. (2020). Altered functional connectivity patterns of insular subregions in major depressive disorder after electroconvulsive therapy. *Brain Imaging Behav.* 14 753–761. 10.1007/s11682-018-0013-z 30610527

[B50] Wang L. Zang Y. He Y. Liang M. Zhang X. Tian L. (2006). Changes in hippocampal connectivity in the early stages of Alzheimer’s disease: evidence from resting state fMRI. *NeuroImage* 31 496–504. 10.1016/j.neuroimage.2005.12.033 16473024

[B51] Wang Y. Zhang Y. Zhang J. Wang J. Xu J. Li J. (2018). Structural and functional abnormalities of the insular cortex in trigeminal neuralgia: a multimodal magnetic resonance imaging analysis. *Pain* 159 507–514. 10.1097/j.pain.0000000000001120 29200179

[B52] Wu H. Sun H. Xu J. Wu Y. Wang C. Xiao J. (2016). Changed hub and corresponding functional connectivity of subgenual anterior cingulate cortex in major depressive disorder. *Front. Neuroanat.* 10:120. 10.3389/fnana.2016.00120 28018183 PMC5159433

[B53] Wu Y. Zhang Y. Liu Y. Liu J. Duan Y. Wei X. (2016). Distinct changes in functional connectivity in posteromedial cortex subregions during the progress of Alzheimer’s disease. *Front. Neuroanat.* 10:41. 10.3389/fnana.2016.00041 27147982 PMC4828463

[B54] Yu M. Sporns O. Saykin A. J. (2021). The human connectome in Alzheimer disease - relationship to biomarkers and genetics. *Nat. Rev. Neurol.* 17 545–563. 10.1038/s41582-021-00529-1 34285392 PMC8403643

[B55] Zhan Y. Ma J. Alexander-Bloch A. F. Xu K. Cui Y. Feng Q. (2016). Longitudinal study of impaired intra- and inter-network brain connectivity in subjects at high risk for Alzheimer’s disease. *J. Alzheimers Dis.* 52 913–927. 10.3233/JAD-160008 27060962

